# Pachychoroid Spectrum Disorders: An Updated Review

**DOI:** 10.18502/jovr.v18i2.13188

**Published:** 2023-04-19

**Authors:** Richard B. Brown, Sashwanthi Mohan, Jay Chhablani

**Affiliations:** ^1^Department of Ophthalmology, University of Pittsburgh School of Medicine, Pittsburgh, Pennsylvania, USA; ^2^Rajan Eye Care Hospital, Chennai, India; ^4^https://orcid.org/0000-0003-2539-2728; ^5^https://orcid.org/0000-0003-1772-3558

**Keywords:** Central Serous Chorioretinopathy, Choroid; Pachychoroid, Focal Choroidal Excavation, Pachychoroid Neovasculopathy, Peripapillary Pachychoroid Syndrome, Peripapillary Pachychoroid Neovasculopathy, Polypoidal Choroidal Vasculopathy, Retinopathy

## Abstract

Pachychoroid disease spectrum is a recent term that has been associated with an increasing number of phenotypes. This review discusses updated findings for each of the typical pachychoroid entities (central serous chorioretinopathy, pachychoroid pigment epitheliopathy, pachychoroid neovasculopathy, polypoidal choroidal vasculopathy, peripapillary pachychoroid syndrome, and focal choroidal excavation), as well as two relatively new additions (peripapillary pachychoroid neovasculopathy and peripheral exudative hemorrhagic chorioretinopathy). Here, we discuss the potential pathogenic mechanisms for these diseases and relevant imaging updates. Finally, we argue for a consistent classification scheme for these entities.

##  INTRODUCTION

Pachychoroid is a relatively recent term that has been used to describe a thickened Haller choroidal layer with attenuation of the Sattler and choriocapillaris layers;^[1]^ however, in the near decade since the term's introduction, a universal agreement on its definition has yet to be established. The result of this lack of consensus in defining characteristics has led some authors to combine disease entities while others separate them in their analyses.^[2]^

Recently, many authors have delineated between pachychoroid and non-pachychoroid forms of pachychoroid disease spectrum (PDS) entities, such as central serous chorioretinopathy (CSCR), pachychoroid neovasculopathy (PNV), and polypoidal choroidal vasculopathy (PCV).^[2,3,4,5,6]^ Notably, Yamashiro et al described that several studies from 2012 to 2018 variably classify PNV (“pachychoroid-driven macular neovascularization without polypoidal lesions”) and PCV (“pachychoroid-driven macular neovascularization with polypoidal lesions”); during this period, cases that would classify as PCV under the current framework were labeled as PNV.^[2]^ Since then, PNV and PCV have tended to be reported as separate entities; however, some authors have continued to include eyes with polypoidal features in PNV.^[2]^ This, therefore, creates issues when reporting imaging findings, treatment outcomes, and clinical management strategies. A detailed discussion of the nomenclature history and issues it has posed can be found in the work by Yamashiro et al.^[2]^ Moving forward, a widespread consensus on the inclusion criteria for pachychoroid spectrum and each pachychoroid entity is paramount to building our understanding of these diseases and their correct pathogenetic mechanism.^[1,2,3]^ Recently, the following diagnosis criteria has been proposed for pachychoroid: (1) reduced fundus tessellation, (2) pachyvessels, defined as dilated choroidal vessels seen on optical coherence tomography (OCT) or indocyanine green angiography (ICGA), extending the entire length of the vessel to the vortex vein ampullae, causing choriocapillaris and Sattler layer attenuation, (3) a lack of soft-drusen (an exception is made for pachydrusen, which are irregular, scattered yellow–white deposits across the posterior pole), and (4) the presence of CSCR characteristics, such as retinal pigment epithelium (RPE) abnormalities, choroidal vascular hyperpermeability (CVH), or a prior CSCR diagnosis.^[2]^

This review argues for a more consistent classification scheme for these entities, focusing on the most updated findings for each: CSCR, pachychoroid pigment epitheliopathy (PPE), PNV, PCV, peripapillary pachychoroid syndrome (PPS), and focal choroidal excavation (FCE), as well as newer entities recently added to the spectrum – peripapillary pachychoroid neovasculopathy and peripheral exudative hemorrhagic chorioretinopathy. Further, this review discusses the updated pathogenesis for these entities and attempts to address the debate that these entities exist on a single disease spectrum. Recent imaging analysis will also be discussed.

##  METHODS

Articles were found by searching online databases for terms such as “pachychoroid” and combinations of the individual entity titles as listed above with “imaging” and “pathogenesis.” Relevant articles published in English language were included in this analysis. Emphasis was given to recently published papers.

##  RESULTS

### Pachychoroid Disease Spectrum Entities 

### CSCR

Central Serous Chorioretinopathy (CSCR) is the classical pachychoroid spectrum entity with a relatively large body of research; despite this, significant questions remain about its pathogenesis and classification criteria. Compared to other pachychoroid disease spectrum (PDS) phenotypes, CSCR presents in relatively younger patients with serous neurosensory retinal detachment, which may accompany pigment epithelium detachments (PED).^[3]^ As Manayath et al describe, CSCR may also present with posterior choroidal fluid loculations.^[7]^

Treatment of CSCR depends on disease severity, functional impairment, and patient preference, among other factors. Acute cases are relatively more likely to resolve spontaneously without visual consequence and can be observed.^[8]^ Treatment may be clinically warranted in patients with existing monocular vision, significant symptomatology, or those who desire it.^[3]^ Chronic cases are more likely to require treatment, given their potential to develop neovascular complications, RPE atrophy, or cystoid macular degeneration.^[3,8,9,10]^ Patients receiving treatment are more likely to have favorable vision outcomes compared to observation alone.^[10,11]^ Half-dose Verteporfin Photodynamic Therapy (vPDT) has become a popular treatment choice given its efficacy and improved safety compared to full-dose vPDT.^[9,12]^ The efficacy of spironolactone and eplerenone in CSCR have also been investigated in randomized control trials, finding mixed results in choroidal thickness reduction and significant change in visual acuity.^[13,14]^ Recently, Kumar Sahoo et al found that sub-foveal vessels were significantly more likely to respond to PDT, while eplerenone significantly decreased central macular thickness and intraretinal cysts.^[10]^ Through multiple meta-analyses, anti-vascular endothelial growth factor (anti-VEGF) treatments provide no significant improvements in visual acuity in CSCR patients;^[15,16]^ however, combination therapy is more useful in the treatment of PDS with neovascular components, such as CSCR with CNV, PNV, and PCV.^[3]^

#### PPE

**Figure 1:**
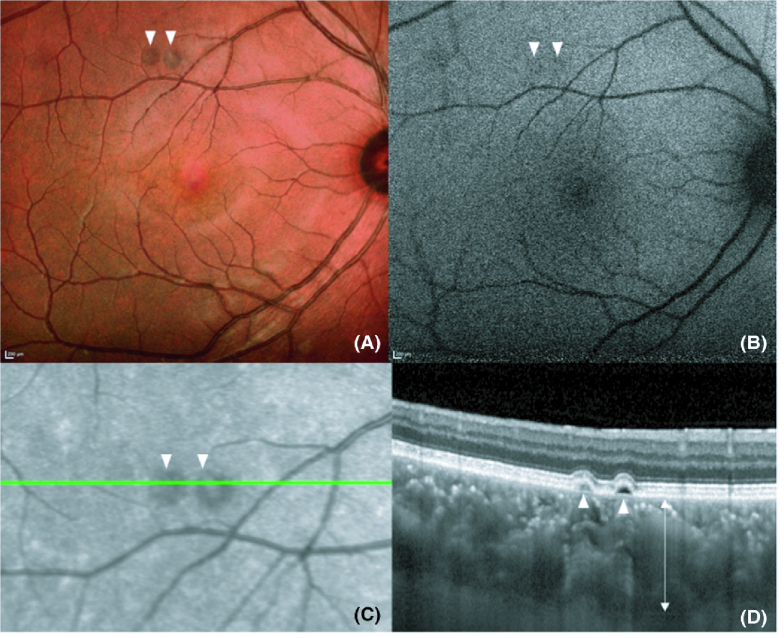
“Imaging of pachychoroid pigment epitheliopathy. (A) Multicolour image highlighting two small PEDs. (B) FAF imaging does not demonstrate major changes in RPE autofluorescence. (C) Near infrared image highlights the position of the line scan for the OCT in image D. Two elevated changes are again seen surrounded by haloes of reduced infrared signal. (D) EDI SD-OCT shows two small serous PEDs (arrowheads) with clear pachychoroid (arrow). PED, pigment epithelial detachment; FAF, fundus autofluorescence; OCT, optical coherence tomography; EDI SD-OCT enhanced depth imaging spectral domain-OCT” [included with permission from Wiley Publishing].^[1]^

Pachychoroid pigment epitheliopathy has been classified into four types: RPE thickening, hyper-reflective RPE spike, RPE elevation with inter-RPE fissures, and PED.^[17]^ Sakurada et al demonstrated decreased choriocapillaris blood flow in areas coinciding with PPE lesions, suggesting that local ischemia may be the basis of this condition.^[18]^ Similarly, Tagawa et al investigated choriocapillaris flow changes in 32 eyes with PPE compared to 30 healthy controls, finding that eyes with PPE had significantly larger mean total flow void area and average flow void size compared to healthy controls.^[19]^ Further, PPE eyes tended to exhibit a diffuse decrease in choriocapillaris blood flow area, not necessarily spatially related to pachyvessel location. Interestingly, only 21.3% of flow void areas were present over a pachyvessel, leading these authors to suggest that pachyvessel presence does not directly result in choriocapillaris flow deficits.^[19]^ A characteristic case of PPE is shown in Figure 1.

**Figure 2:**
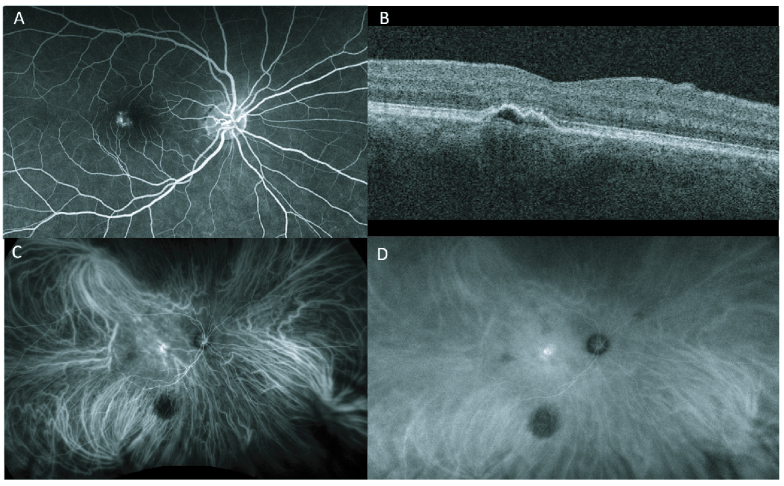
A 58-year-old female with PNV in the right eye. (A) FA demonstrating hyperfluorescence with minimal leakage. (B) OCT showing pigment epithelial detachment (PED) with hyperreflectivity within PED. (C) Early ICG which depicts dilated choroidal vessels and hyperfluorescence. (D) ICG late phase shows diffuse choroidal fluorescence with increasing hyperfluorescence at the lesion site.

**Figure 3:**
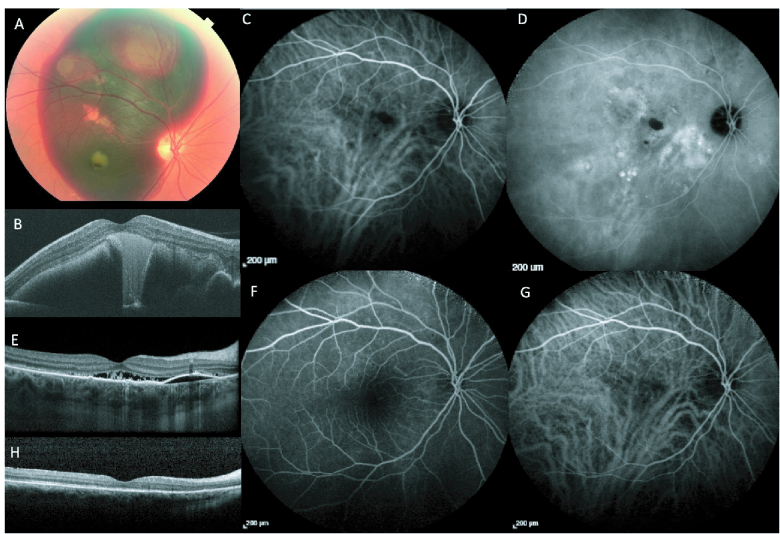
Images of Pachychoroid Choroidal Vasculopathy in a 59-year-old male. (A) Color fundus imaging demonstrating a large, elevated subretinal hemorrhage involving majority of macula and extending to superior mid-periphery. (B) OCT imaging at case presentation. The patient underwent pars-plana vitrectomy, subretinal TPA, SF6, and Aflibercept injection. ICGA post-surgery, early (C) and late (D) stages, showed multiple focal leaks along with polypoidal network. OCT at this stage (E) showed subretinal fluid and pigment epithelial detachment. Subsequently, half-fluence PDT was performed, and complete regression of network was achieved, as shown on early (F) and late (G) phases of ICGA and no activity on OCT (H).

**Figure 4:**
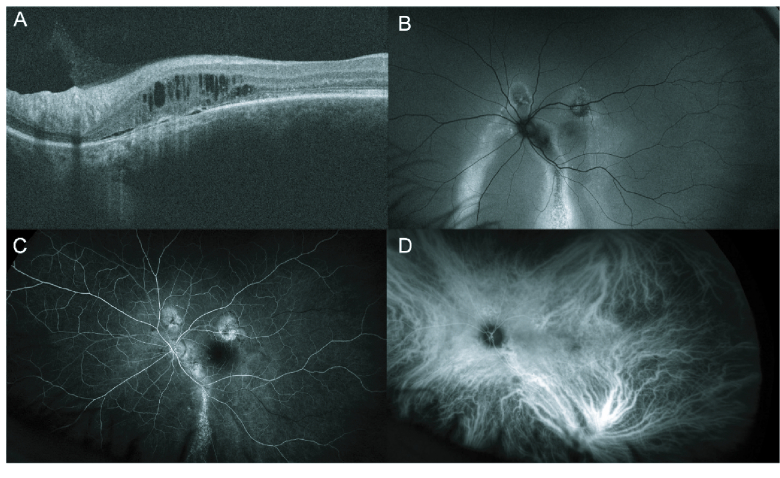
Images of Peripapillary Pachychoroid Syndrome in a 45-year-old male. OCT image (A) demonstrating peripapillary intra-retinal fluid accumulation, with minimal sub-retinal fluid. Fundus autofluorescence (B) shows hyper- and hypoautofluorescence changes along with retinal pigment epithelial tracts. Fluorescein angiography (C), and indocyanine green angiography (D) demonstrate choroidal vascular dilation, along with multiple areas of hyperfluorescence.

**Figure 5:**
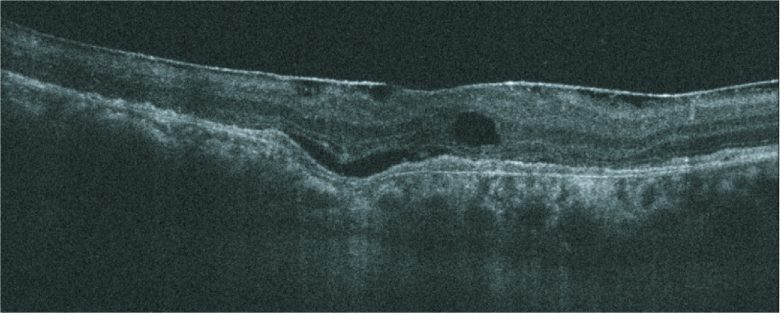
Optical coherence tomography of a 69-year-old male with CSCR demonstrating Focal Choroidal Excavation, along with intra-retinal fluid and an epiretinal membrane.

**Figure 6:**
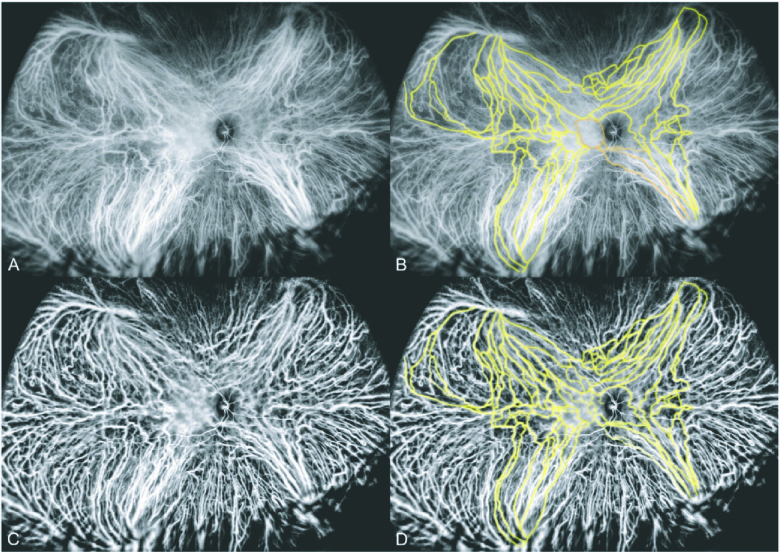
“Wide-field ICG angiogram of a 48-year-old with central serous chorioretinopathy. (A) Image obtained 2:51 min after indocyanine green dye injection. (B) Some of the intervortex venous anastomoses are highlighted in yellow for easier recognition. There are a group of vessels, highlighted in orange, that lead to the inferior macular region and are no longer visible in the central macula because of early leakage (area surrounded by orange outline). (C) Using wavelet contrast enhancement of the middle and low spatial frequency components, haze was removed from the image leaving clear delineation of the larger vessels. (D) The central intervortex venous anastomoses are visible, as highlighted in yellow” [included with permission from Elsevier.]^[8]^

#### PNV

There has been much debate in the literature regarding the classification and nomenclature of pachychoroid neovascularization (PNV). Previous studies utilized a variety of diagnostic criteria for PNV based on imaging findings, clinical characteristics, and choroidal thickness, among other elements,^[20]^ making comparisons between studies challenging.^[2]^ Such ambiguity in inclusion criteria yielded subjective inclusion of polypoid lesions in PNV studies; accordingly, Yamashiro et al further proposed that moving forward PNV specifically exclude pachychoroid-driven macular neovascularization with polypoidal lesions, as these should tentatively be termed PCV.^[2]^

Etiologically, PNV joined the pachychoroid spectrum after imaging demonstrated that neovascular lesions spatially correspond to areas with common pachychoroid features, such as thickened choroid, pachyvessels, and increased CVH.^[1,3,20]^ Some investigators have suggested that PNV is a late complication of preexisting PPE or chronic CSCR^[1]^; however, this link is contentious.^[21]^ In support of this theory, previous authors have suggested that given the extensive metabolic demand of photoreceptor cells and relatively limited oxygen supply of the inner retinal vasculature, choroidal thickening decreases diffusion of available oxygen supply from the choriocapillaris to the outer retina, and may therefore lead to VEGF expression from the RPE and subsequent neovascularization.^[1]^ Conversely, others have argued that PNV and PCV occur through mechanisms distinct from the uncomplicated pachychoroid-PPE–CSCR pathway. Demirel et al found that central choroidal thickness (CT) and choroidal vascularity index (CVI) were significantly different between PNV & PCV, PNV & CSCR, and CSCR & PCV, while there was no difference between CT and CVI in PPE & PNV and PPE & CSCR.^[21]^ These authors argued that if PNV and PCV are continuations of the CSCR pathogenesis, these cases should have comparable CVI values to CSCR eyes, rather than the relatively lower CVI reported both in their study and elsewhere in the literature.^[21,22]^ Although other authors have explained the decreased CVI in PNV by suggesting that anastomoses may relieve choroidal congestion, thereby decreasing choroidal thickness and prompting changes in CVI,^[23]^ the direct link between CSCR-related entities and PNV/PCV has still not been causally related.^[21]^ Further studies elucidating the relationships between these entities are still needed.

In addition, much debate has centered around the involvement of neovascular age-related macular degeneration (nAMD) lesions in PNV classification structures.^[2,24]^ Some authors have suggested using neovasculature patterns as a differentiation tool.^[20]^ Others have divided PNV cases based on the presence or absence of irregular PED legions.^[25]^ Patients with PNV also tend to be younger and have more RPE abnormalities, thicker choroids, and minimal/absent drusen (excluding pachydrusen) compared to nAMD.^[1,3,26]^ A distinction between drusen-driven AMD and PNV has been supported with deep machine learning artificial intelligence^[27]^ and with cluster analysis.^[28]^ Further, the inflammatory cytokine profile in eyes with PNV differs from those with nAMD.^[29]^ Previous studies also suggested eyes with PNV may have lower VEGF concentrations compared to nAMD^[30]^; however, VEGF compartmentalization within the choroid may complicate quantitative measurements.^[5,20]^

Advances in imaging techniques are also thought to be important in PNV differentiation [Figure 2]. Pulsation seen in the downstream of the vortex vein on ICGA has recently been proposed as a biomarker for choroidal overload, particularly in eyes with PNV.^[31]^ Conversely, optical coherence tomography angiography (OCTA) has shown a higher sensitivity and specificity than dye angiography in PNV identification.^[3,32]^ Specifically, cases with type 1 CNV and shallow irregular PEDs are better detected on OCTA imaging,^[3,33]^ and small PNV lesions with indistinct capillary patterns, fewer core vessels, or absent plaque hypercyanescence are all significantly more likely to be missed by dye angiography.^[32]^ Recently, Sagar et al proposed that SS-OCT may be the most helpful in diagnosing and determining the treatment efficacy.^[20]^ PNV has been shown to exhibit shallow, irregular PEDs on OCT, while the lesions in PCV are sharper and notched.^[20]^ Such irregular PEDs also correspond to the RPE-BM neovascularizations seen on OCTA.^[20]^

Many treatment options have been explored for PNV; however, differences in case inclusion between studies somewhat cloud this work.^[2]^ Anti-VEGF treatments are equally effective in both PNV and nAMD, but PNV patients often require a longer treatment interval.^[3]^ Aflibercept has proven more effective than ranibizumab at targeting CVH, reducing fluid, and decreasing choroidal thickness.^[20,34,35]^ Effects of full-fluence and half-fluence PDT have also been evaluated.^[36,37]^ PNV and chronic CSCR have recently been shown to respond differently to half-fluence PDT treatment and may require anti-VEGF + half-fluence PDT combination therapy.^[37]^ Plausibly, PDT monotherapy may successfully treat the CVH component of PNV; however, the neovascular lesions still require an anti-VEGF medication.^[36,37]^ Monotherapy with anti-VEGF injections^[34]^ or full-fluence PDT,^[36]^ as well as combination therapy,^[38]^ have led to the need for retreatments; however, the lowest recurrence incidence (19%) has been in half-dose PDT combined with aflibercept injections.^[39]^ Overall, the PDT + Anti-VEGF regimens are well tolerated; this combination may result in fewer needed injections and reduce retreatment burden.^[1,38]^ Future prospective trials are needed to create definitive guidelines surrounding functionally effective treatment modalities for these patients.

#### PCV/AT1/PAT1

Polypoidal choroid vasculopathy (PCV) has been described as polypoidal vascular dilations overlying a choroidal vascular network.^[3]^ PCV carries many of the same features as PNV, including type 1 (sub-RPE) neovascularization between the RPE-Bruch's membrane junction corresponding to pachyvessel and CVH location.^[1,3]^ PCV has also been associated with serous or hemorrhagic PEDs near the polypoidal legions [Figure 3] and sub- or intraretinal exudations.^[1,40]^ Some recent authors suggest that EDI-OCT is crucial in diagnosing this entity, which may demonstrate peaked PEDs containing “thumbprint signs,” or rings of hyperreflectivity with hypo-reflective lumens.^[41]^ A “double hump sign” may present where one PED leaks fluid near another PED, while saccular dilations may appear as hyperreflectivity at the RPE level on enface OCT.^[41]^ On imaging, OCTA is more sensitive than ICGA in detecting type 1 macular neovascularization and may help both identify PCV cases and differentiate them from other PDS entities and nAM.^[32,42]^ Interestingly, the aneurysm-like structures in PCV/AT1 are theorized to result from exposure to pulsatile blood flow,^[43]^ and previous work demonstrated that pulsatile lesions were significantly more likely to be missed on OCTA compared to ICGA.^[44]^

A wide variety of terms have been used to describe this combination of clinical features and have been reviewed elsewhere.^[2,45]^ These authors note differences in the frameworks used to classify macular neovascularization, which have compromised the clinical utility of previous works.^[2]^ Recently, aneurysmal type 1 neovascularization (AT1) or pachychoroid aneurysmal type 1 neovascularization (PAT1) have become common in the literature, removing the “polypoidal” terminology to reflect the idea that the lesions are primarily vascular rather than epithelial.^[1,3][46]^ A consensus-driven, universal framework is paramount to future studies and our comprehension of the disease. Accordingly, Yamashiro et al recommended that the term PCV should be used cautiously until we have a concreate understanding of the pathogenetic mechanism.^[2]^

PCV has been proposed to be a common clinical manifestation of several pathogenic processes.^[2,5]^ PCV and nAMD have similar features;^[47]^ genetic overlap between PCV/AT1/PAT1 and nAMD has been discussed previously.^[1,48]^ PCV tends to present in younger patients with greater choroidal thickness, CVH, and RPE defects, in comparison to the thinner choroids seen in nAMD.^[3]^ Further, both pachychoroid- and non-pachychoroid-PCV have been discussed.^[4,49]^ Pachychoroid-PCV and CSCR are more likely to demonstrate diffuse pachyvessels, while in non-pachychoroid-PCV and AMD eyes pachyvessels tend to be focal.^[49]^ Ultimately, Yamashiro et al recommended a framework in which drusen-driven disease entities are classified as nAMD with or without polypoidal lesions, while diseases without drusen are termed “PNV with polypoidal lesions” or “PNV without polypoidal lesions.”^[2]^

Many previous studies do not specify the PCV subtype of their subjects, causing ambiguity when interpreting their results.^[5]^ Pachychoroid-PCV eyes are more likely than non-pachychoroid-PCV to have persistent retinal fluid^[4]^ and chronically increased SFCT after anti-VEGF monotreatment.^[6]^ Further, following anti-VEGF monotherapy, BCVA does not significantly differ from baseline to 60-month follow-up in either group, or between groups.^[6]^ Recently, Vadalà et al evaluated full fluence vPDT + aflibercept in pachychoroid-PCV eyes, finding significant improvement in functional outcomes at 12 months;^[5]^ these findings are similar to those previously reported.^[50]^ Initially treating with vPDT before aflibercept injections has been hypothesized to maintain the effects of initial vPDT and reduce retreatment in PCV and PNV patients.^[5,38]^

Previous authors have speculated that baseline choroidal thickness may be an important predictor for final vision outcomes.^[3,51,52]^ Although Chang and Cheng proposed a sub-foveal choroidal thickness (SFCT) threshold of 267.5 µm for anti-VEGF treatment efficacy,^[4]^ the clinical utility of SFCT thresholds is controversial.^[53]^ Patients with PCV may also exhibit significant variability in choroidal thickness,^[3,54]^ making threshold applications challenging for clinical diagnosis and treatment selection. Further, given the similarity in functional outcomes reported by Shimizu et al,^[6]^ it is unclear if choroidal thickness thresholds will hold significant clinical value in these cases.

#### PPS

Peripapillary pachychoroid syndrome (PPS) is a relatively new addition to the pachychoroid spectrum, with few cases reported overall.^[55]^ In PPS, patients demonstrate intra or subretinal fluid in the region nasal to the macula and choroidal thickening near the optic disk, rather than the fovea.^[3,56]^ The disease tends to occur in elderly males^[1,56,57,58,59]^ and may present bilaterally.^[3,56][60]^ Patients with this condition may also have other features of PDS, such as CVH, pachyvessels, pigment epitheliopathy, serous PEDs, and hyperfluorescence in the peripapillary region [Figure 4].^[3,41]^ PPS also exhibits choroidal folds, smaller cup-to-disk ratios, and mild disk leakage on late FA.^[41,56]^ Recently, Barequet et al described cases of acquired vitelliform lesions in eyes with PPS, a new finding for this disease entity.^[59]^

The etiology of PPS is still contentious. Some authors have speculated that PPS may have a similar pathogenesis as CSCR, wherein papillary choroidal congestion results in RPE dysfunction and subretinal fluid accumulation.^[55,56]^ Some have proposed that fluid from the nasal choroid may leak into the retinal space via membrane defects in the external limiting membrane (ELM),^[55]^ while others purport that the peripapillary fluid pocket signifies the fluid entry site from the choroidal vasculature into the retina.^[60]^ Recent evidence has led authors to theorize that anastomotic connections may also play a role in PPS pathogenesis.^[43,55]^ Peripapillary anastomoses, in combination with the lack of RPE around the optic nerve head, may allow for the direct transmission of choroidal hydrostatic pressure to the inner retinal layers in this region.^[57]^

Treatment efficacy for PPS varies. Recent cases have resolved spontaneously^[55]^ or with PDT laser.^[60]^ A case of a bilateral PPS, initially treated with dorzolamide eye drops and eplerenone tablets, ultimately achieved retinal fluid resolution and visual acuity improvement after treatment with dexamethasone drops.^[60]^ In both Bouzika cases, visual acuity was significantly affected, making treatment a necessity over the observation only modality suggested by other authors.^[55]^

The efficacy of anti-VEGF injections is mixed; some have reported favorable outcomes after aflibercept injections,^[61]^ while others reported cases of PPS that were recalcitrant to multiple anti-VEGF medications.^[56,62,63]^ Recalcitrant cases have recently shown improved BCVA after low-fluence PDT.^[63]^ Investigations of PDT have also shown long-term significant decreases in both SRF and CSFT,^[58]^ but with complete resolution in fewer patients than seen in CSCR populations.^[64]^ Xu et al investigated long-term treatment outcomes and found that eyes receiving anti-VEGF injections exhibited decreases in choroidal and retinal thickness, but no significant change in best-corrected visual acuity (VA). However, several eyes in this study had remaining SRF which may contribute to the lack of BCVA gain, or had photoreceptor atrophy, which would portend poor vision outcomes regardless of fluid resolution.^[57]^

Most recently, some reports have documented the potential utility of topical steroids in these cases with good anatomical outcomes.^[65,66]^ Accordingly, topical steroids can be used as a treatment for fluid resolution PPS; however, because treatment may require extended use and may cause other ocular complications associated with elevated IOP, this modality may be best reserved for recalcitrant cases.

#### FCE

Focal choroidal excavation (FCE) presents as a choroidal concavity seen on OCT^[67]^ in patients without scleral ectasia, posterior staphyloma, or other scleral abnormalities [Figure 5].^[3,20]^ Given its choroidal thickening, CVH, and dilated pachyvessels, FCE has been linked with PDS.^[68]^ The place of FCE within the spectrum has not been delineated. FCE coincidence has been documented in a small percentage of CSCR and PCV/PAT1 cases.^[69,70,71]^

Few known risk factors exist; FCE cases have no gender predisposition and affect a wide range of ages.^[1,53,70]^ Although FCE usually presents unilaterally and within the fovea, some recent reports suggest it may rarely manifest extrafoveally.^[72]^ FCE is difficult to detect on fundus exam alone but may occasionally present as small yellow lesions and pigmentary changes.^[41]^ EDI-OCT can be useful during diagnosis, as it can demonstrate pachyvessels enlargement, choriocapillaris atrophy, and an intact, depressed RPE.^[41]^ On OCT and ICGA, these cases also tend to demonstrate choroidal attenuation directly below the excavation area corresponding to outer choroidal vessel dilation.^[73]^

Several classification systems of FCE have been described in the literature. Margolis et al presented “conforming FCE” and “non-conforming FCE,” where conforming indicates an intact photoreceptor-RPE junction, while non-conforming signifies a hypo-reflective space between these layers.^[74]^ Later, Shinojima et al proposed three morphological shapes for FCE: bowl, cone, or mixed type, depending on their appearance on OCT.^[71]^ Seemingly, bowl-shaped patterns correlate with more significant atrophic changes and may be associated with a worse prognosis.^[71]^ In a recent study, Capellan et al described three subtypes based on thresholds of central choroidal thickness.^[75]^ These authors found that FCE cases presenting with increased thickness ( > 200 μm) and cone-shaped morphology were significantly more likely to develop CNV membranes.^[75]^ Finally, the classification structure proposed by Verma et al includes both congenital and acquired FCE, wherein acquired FCE can occur due to a variety of causes, including inflammation, dystrophy, malignancy, or pachychoroid diseases.^[67]^

Overall, the etiology of FCE is still debated. Some authors have suggested these lesions may arise from a congenital defect in the choroid.^[74,76]^ Here, the youthful retinal elasticity allows RPE conformity to the area of the predisposing choroidal malformation,^[77]^ further permitting the photoreceptor-RPE junction to remain intact and preventing visual manifestations.^[74]^With aging, however, decreased elasticity results in layer separation and photoreceptor ischemia, which ultimately cause clinical symptoms and prompt presentation.^[67]^ Other investigators have suggested the disease can also be acquired, with some proposing that inflammation, fibrosis, CNV, or choriocapillaris atrophy may weaken the junction between the RPE and BM, decreasing the overall architectural support and leading to FCE development.^[67,73,78]^

Recently, many authors have discussed inflammatory etiologies of FCE. Verma et al described that hyporeflective spaces between the RPE and photoreceptor layer may also contain hyperreflective elements, potentially indicating residual inflammation or outer segment photoceptor degeneration.^[67]^ Similarly, Ellabban et al suggested that hyperreflective tissue underlying the legion may be evidence of a previous choroidal scar tissue, contraction of which may generate the FCE lesion.^[69]^ Potentially, in cases of severe inflammation, adhesions between the layers may result in a conforming lesion, whereas cases of mild inflammation may result in less adhesion and layer non-conformity.^[67]^ Gan et al found that a majority of FCEs form nearby other comorbid retinal lesions, either at locations within the comorbidity or at its edges, prompting them to suggest that FCEs may occur following other pathologic retinal alterations.^[78]^

Further, the connection between disease etiology and potential prognosis remains unclear. Verma et al suggested that congenital FCE tended to be static and non-vision threatening, while acquired FCE had the potential to cause complications and vision loss.^[67]^ In 2017, Chung et al noted that lesions tend not to change over time;^[68]^ however, in the study by Gan et al, 22.5% (14 eyes) of eyes with comorbidities experienced a pattern change while 4.8% (three eyes) developed neovascular lesions.^[78]^ If these lesions were acquired secondarily to the underlying disease or clinical treatment, these findings are congruent with Verma et al, who stated that acquired cases tend to have higher complication rates. Large-scale, prospective studies describing the natural course of pachychoroid FCE are needed to validate various classification systems, which may have prognostic value in assessing complication risk and determining clinical follow-up interval.^[75]^ Regular observation is recommended to monitor for treatment-requiring complications, such as neovascularization. To date, no case reports or studies in the literature suggest the need to alter the standard treatment course for the coexisting conditions.^[67]^

### New entities

Recently, several new entities have been proposed to add to the pachychoroid spectrum.

#### Peripapillary pachychoroid neovasculopathy

Montero Hernandez et al presented a new entity in the pachychoroid spectrum, peripapillary pachychoroid neovasculopathy (PPN), which describes PPS occurring with peripapillary type 1 CNV.^[79]^ Notable findings included: papillonasal pigmentary changes overlying mottled autofluorescence; an irregular PED and pachyvessels on SD-OCT; a large neovascular network and hyper-flow signal on OCTA; and CVH on ICGA. The patient was given treat-and-extend aflibercept, resulting in a good visual and anatomic outcome.^[79]^

#### Peripheral exudative hemorrhagic chorioretinopathy

First described by Annesley in 1980, Peripheral exudative hemorrhagic chorioretinopathy (PEHCR) is associated with peripheral subretinal fluid and hemorrhaging, typically located between the ocular equator and ora serrata.^[80]^ Imaging studies have found polypoid lesions in the retinal periphery on ICGA, prompting authors to suggest it is similar to PCV.^[81,82]^ Schroff et al recently proposed PEHCR as an addition to the pachychoroid spectrum after finding that it was associated with a thickened choroid in the temporal periphery.^[80]^ This pattern of gradually thinning toward the fovea is the inverse of both CSCR ^[83]^ and normal eyes,^[84]^ which are thickened subfovealy. This entity has been previously thought to be a variant of A^[85]^ however, AMD demonstrates a relatively thinner retina, leading these authors to suggest it is a separate entity with a different pathogenetic mechanism.^[80]^ Mantel et al described these cases as being self-limiting, with long-term follow-up demonstrating stability, regression, or full resolution.^[81]^ Importantly, given the similarities in their presentation, these lesions must be distinguished from choroidal melanoma which may prevent unneeded radiation or enucleation in these patients.^[86]^

#### Pathogenesis

The pathogenetic mechanism of pachychoroid entities is perhaps the most heavily debated component of these conditions. Seemingly, authors agree that uncomplicated pachychoroid, PPE, and CSCR represent stages of a single pathologic entity. However, some disagree that other PDS entities exist on the same linear spectrum.^[21]^ To date, the collective findings suggest that CSCR eyes experience reduced choriocapillaris blood flow spatially corresponding to areas of retinal pigment epithelium ischemia and dysfunction.^[87]^ They propose that the pachyvessel enlargement in eyes with uncomplicated pachychoroid may damage the RPE, leading to PPE, which then becomes CSCR after the RPE damage becomes so significant that it cannot compensate for fluid accumulation and leads to SRF formation.^[21]^

Generally, authors agree that the fluid in CSCR results from CVH; however, the inciting factors for this CVH are still highly contentious.^[8]^ Importantly, conditions in the PDS spectrum exhibit signs of venous overload,^[88]^ which has been suggested by many as the underlying cause of CVH.^[8,43,89,90]^ Without compensatory lymphatic vessels,^[91]^ the retina relies on the choriocapillaris and RPE to remove excess fluid from the subretinal space; such elevated venous pressure prevents reabsorption and leads to fluid pooling.^[8]^

Increased venous pressure has been theorized to result from increased blood flow or decreased venous emptying.^[8]^ Some have proposed choroidal arteriovenous malformations or fistulas cause arterial blood to flow directly into a choroidal vein; this increased pressure from arterial blood may cause venous dilation and congestion, leading to pachyvessel formation.^[90]^ In this framework, an anastomotic connection between choroidal arteries and vortex veins results in CSCR, while a connection between a choroidal artery and a choroidopial vein results in PPS.^[90]^

Separately, increasing evidence has suggested that abnormal venous emptying via vortex vein occlusion may be responsible for choroidal venous congestion and CVH.^[43,92,93,94]^ Kishi and Matsumoto described ICGA studies in CSCR eyes which revealed a dilation of the vortex vein ampulla, suggesting an obstruction where the vortex vein transverses the sclera;^[43]^ other authors have hypothesized this site may hold a valve-like function regulating venous outflow.^[8,94]^ Further, eyes with CSCR and PNV have increased scleral thickness,^[95]^ which may increase outflow resistance^[94]^ and decrease diffusion permeability,^[8]^ contributing to venous stasis and congestion. In addition, risk factors associated with CSCR development have also been associated with increased scleral thickness. CSCR is more common in middle-aged men,^[8]^ who have a relatively thicker sclera than women.^[96]^ Hyperopic eyes also tend to have thicker anterior sclera, suggesting that axial length may be an intrinsic risk factor for CSCR development.^[43,97,98]^ Further studies are needed to understand the contributions of scleral thickness and rigidity to overall vascular resistance.^[43]^

Vortex vein asymmetry may also contribute to venous congestion in CSCR eyes.^[99]^ Pachyvessels tend to adhere to a specific vortex vein quadrant.^[3,88,90]^ Further, PDS eyes demonstrated significant inter-subject variability in the fundal proportion drained by each vortex vein, which was not seen in control eyes.^[94]^ Underdevelopment of one vortex system may represent anatomy predisposed to choroidal venous congestion, overload, and development of PDS.^[94]^

In addition, venous congestion and stasis have been reported to induce expression of cytokines and other signaling molecules that cause venous dilation and vasculature remodeling.^[8]^ Kishi and Matsumoto suggested that acute venous stasis causes asymmetrical vein engorgement in an area which spatially corresponds to choriocapillaris filling delays; over time, compensatory anastomoses form to relieve chronic venous congestion.^[43]^ Multiple studies have found an association between inter-vortex venous anastomotic connections and pachychoroid entities.^[23,89,93,100]^ Spatially, eyes with CSCR and PNV tended to have anastomoses in the central macula, while eyes with PPS had anastomoses near the optic nerve, providing evidence of a common link between these phenotypes.^[89]^

Seemingly, these anastomotic vessels have relatively thin walls, making them susceptible to the elevated venous pressure that exists secondary to the venous stasis in these diseases. Dilation of these predisposed vessels were thus purported as the pathogenesis of pachyvessels.^[43]^ Interestingly, as one choroidal system forms a venous anastomosis to relieve congestion and stasis, other authors have suggested that the neighboring system may already have relatively greater venous congestion, thereby, potentiating the venous overload and choriocapillaris leakage.^[8]^ Spaide et al also purported that these dilated anastomoses transmit increased venous pressure to the choriocapillaris, resulting in both leakage and structural damage; therefore, the choriocapillaris attenuation extensively described by previous authors may be due to venous flow abnormalities than mechanical choriocapillaris compression.^[8,89,92]^ Further, in some cases, the anastomoses became the prominent vessels within a region, crowding out normal choroidal veins until none remained.^[89]^ Similarly, analyses of the choriocapillaris in eyes with CSCR and PPS found an overall decrease in capillaries, with the ones that remained being longer and wider than normal controls.^[8,92]^ Chen et al occluded the vortex veins in monkeys, initially noting increased choroidal thickness in the distribution of the occlusion, however, after three months found that non-occluded regions were also thickened, potentially suggesting anastomoses formation resulted in diffuse vortex vein involvement.^[101]^ Overall, it is unclear if vein-to-vein anastomoses occur as a result of venous obstruction, as suggested by Kishi and Matsumoto,^[43]^ or caused by arterio-venous anastomoses, as suggested by Brinks et al.^[90]^ Future studies need to be conducted to delineate if anastomoses are dilations of existing vessels or a neovascular consequence of these pathologies [Figure 6].^[8]^

Interestingly, Bacci et al found that intervortex venous anastomoses and CVH also occur in healthy eyes, which they attributed to potential subclinical forms of venous insufficiency.^[94]^ Similarly, Jeong et al quantitatively evaluated the choroidal vasculature patients with unilateral CSCR, finding no significant difference in vortex vein engorgement in affected and unaffected eyes, providing further evidence that a predisposition may be present in these patients.^[93]^ Shinojima et al found that 19% of asymptomatic/contralateral eyes of patients with unilateral CSCR ultimately developed a retinal detachment during the follow-up period, further suggesting there may be a predisposing factor.^[102]^ Gerardy et al found that the asymptomatic eyes of unilateral CSCR cases exhibited significantly reduced foveal cone density, suggesting that photoreceptors may be damaged at baseline in a process independent of retinal detachment; these cones may be particularly sensitive to oxidative stress or abnormal blood flow regulation, which is congruent with findings that CSCR patients exhibit higher levels of oxidative stress biomarkers.^[103]^ Kim et al recently investigated the characteristics surrounding RPE detachment location in CSCR patients.^[104]^ They noted that PEDs closer to the foveal center tended to develop CNV, leading them to hypothesize that the foveal RPE in CSCR patients may be more sensitive to hypoxia, RPE insufficiency, or other tissue stressors.^[104]^

PPE transitioning to other pachychoroid entities have been reported, including CSCR,^[17,105]^ PNV,^[105]^ and PCV,^[106,107]^ which suggests that these entities are related and may be stages of a single disease process.^[1,3]^ The contralateral, asymptomatic eyes of unilateral CSCR patients have shown signs of PPE in the majority of cases.^[105,108]^ Similarly, patients with unilateral PCV have also demonstrated signs of PPE in their contralateral eye, all of which became PCV over time.^[107]^ These eyes may have more severe choriocapillaris thinning, leading to further ischemia and RPE disruption.^[107,109]^ Tang et al further hypothesized that the RPE irregularities seen in PPE represent focal origins to PCV lesions.^[107]^ Eyes with an SFCT of < 300 µm may represent very early-stage, compensated PPE.^[105]^

Recently, authors have suggested that acute and chronic CSCR may have different pathophysiologic mechanisms. Acute CSCR may be due to single anastomosis that spontaneously occludes, prompting fluid resolution, whereas chronic cases may involve multiple anastomoses which involve greater retinal surface area.^[90]^ This hypothesis is congruent with the clinical findings of chronic CSCR appearing as broad and shallow compared to acute cases.^[3]^ Imamura et al previously found that the choroid remains abnormal in eyes with resolved CSCR.^[110]^ Some authors suggest that the dilation and hyperpermeability that accompany venous engorgement are permanent changes to the vasculature, particularly in Haller's layer, creating a predisposition for those with acute CSCR to experience fluid recurrence even after episode resolutions.^[8,20,43]^ Chronic CSCR cases have been found to have more hyperpermeable area than acute cases, but without a difference in subfoveal choroidal thickness.^[93]^ Given that patients with acute CSCR have been found to be about 15 years younger than patients with chronic CSCR,^[43]^ some have suggested that older eyes with chronic CSCR may no longer be able to compensate for the fluid overload.^[3]^

Given the affinity of PDT to target dysfunctional endothelial cells, if aberrant anastomoses are involved in CSCR pathogenesis, this may explain the efficacy of PDT as a treatment modality. Studies have suggested that vPDT may induce choroidal vascular remodeling.^[63,111,112]^ By occluding the involved anastomosis, PDT would remove the shunt that is contributing to overload and hyperpermeability.^[90]^ Recent analysis has suggested that although PDT can stop the choroidal leakage, the underlying venous outflow obstruction still remains.^[8]^ Spaide et al discussed surgical decompression of the vortex vein, or the creation of scleral windows to increase scleral outflow; however, the risk profile of PDT likely presents a better option for CSCR patients.^[8]^

Several extrinsic factors may also contribute to CVH and venous overload, resulting in increased choroidal thickness. Systemic steroid use, “Type A” personality, elevated psychological stress levels, and sympathetic over activity have been previously described as risk factors of CSCR.^[113,114,115,116]^ Situations of stress-induced sympathetic response have been hypothesized to increase choroidal blood flow and exacerbate vortex vein stasis.^[43]^ Recently, adrenaline injections in monkeys have shown to cause dilation of the choriocapillaris and choroidal veins.^[116]^ Given these findings, some have suggested that parasympathetic activity may be in some ways protective from CSCR.^[117]^ CSCR eyes have a significant decrease in accommodative ability, which may be linked to a decreased parasympathetic activity in affected eyes.^[117]^ Similarly, pilocarpine, a topical cholinergic, causes choroidal thinning in healthy eyes,^[118]^ while topical atropine, a parasympathetic inhibitor, significantly increases choroidal thickness,^[119]^ providing some support for the hypothesis that sympathetic-parasympathetic dysregulation may lead to a pachychoroid predisposition.^[117]^

#### Imaging 

Previous reviews have detailed choroidal imaging in eyes with PDS diseases;^[1,41,53,87,120]^ clinical findings of these entities are presented there in greater detail. In recent years, advances in choroidal imaging technology and post-acquisition image processing have provided more objective biomarkers for disease identification and treatment efficacy. The focus of this section will be on the utility of parameters and imaging modalities.

Some investigators have argued against absolute quantitative thresholds for choroidal thickness, given that these values may vary with a variety of factors in both diseased and healthy eyes.^[3]^ Choroidal vascular index (CVI), the ratio of vascular lumen to total subfoveal choroid area, has been proposed as a consistent, repeatable measure to monitor disease progression.^[121,122]^ Recently, cluster analysis was used to determine salient criteria for CSCR or PPE differentiation from healthy eyes.^[123]^ Using an unsupervised machine learning technique, these researchers found that the Haller ratio (Haller layer thickness divided by choroidal thickness), choroidal thickness, and CVI were the most important factors for delineation. The Haller ratio was the most valuable single factor, and, along with total choroidal thickness, it was noted that these two values may be the most useful for clinical practice.^[123]^

Pachychoroid-related conditions are thought to have differential blood flow between choroidal layers.^[87]^ Blood flow changes precede the retinal changes seen in these diseases, drawing much attention to imaging techniques.^[49,87]^ EDI-OCT penetrates to the deeper layers of the choroid, allowing for high-resolution imaging and precise quantitative analysis.^[87]^ Further, Swept-Source OCT (SS-OCT) combines the benefit of longer wavelengths (1050 nm) for greater tissue penetration with the rapid capture of numerous images to greatly improve image quality. Averaging multiple images increases in the signal to noise ratio and improves in final image resolution, allowing for both quantitative and qualitative analyses of the choroid.^[87]^ SS-OCTA can also provide clinically relevant information about choriocapillaris flow deficits, as well as identify type 1 and 2 CNV lesions.^[87]^ Variable interscan time analysis (VISTA) has also been combined with SS-OCTA to measure relative blood flow velocity in the choroid, which may be useful in tracking vascular response to treatment over time.^[87]^ Tagawa et al recently demonstrated that choriocapillaris meshwork structure could be visualized *in vivo* by averaging en face OCTA images.^[19]^ Additionally, Doppler imaging has been used to quantify the choriocapillaris and evaluate properties of its blood flow.^[87]^ Future studies utilizing this imaging technique may provide further insight into the various implications of blood flow alterations in these diseases and their respective treatment modalities.

Singh et al further discussed that segmentation slabs on OCT and OCTA machines can help standardize imaging techniques and allows for repeatable choriocapillaris measurements.^[87]^ However, the irregular surface of the inner choroid may cause errors in the automated segmentation process, which may cause inaccurate estimates of flow deficits. Further, RPE atrophy may increase reflectance and cause a mislabeling of normal vessels as CNV.^[87]^ The time involved in manual segmentation or verification is clinically prohibitive.^[87]^

Although much work has been done to report outcomes from therapies, differences in inclusion criteria and mixed findings between studies indicate the requirement for future works to utilize universal criteria in their analyses to clarify past findings. As we learn more about the disease etiology and increasingly agree on an accurate nomenclature system, additional outcome studies may help evaluate the comparative treatment efficacies within specific subgroups. Additional RCTs with long-term follow-up for pachychoroid-PCV specifically would be helpful in this area.

##  Summary

Retinal and choroidal imaging have resulted in major changes to our understanding of pachychoroidal disease entities, including potential mechanisms for their pathophysiology and etiology. Advances in imaging techniques are crucial to delineating the differences between the disease entities on this spectrum and provide clarity surrounding the pathogenesis of pachychoroid subtypes, as well as allow for more precise diagnosis and treatment monitoring. A standardized, consensus-based definition of inclusion criteria is needed to effectively compare future findings and correctly categorize pachychoroid entities.

##  Financial Support and Sponsorship

None.

##  Conflicts of Interest

None declared.

## References

[B1] Borooah S, Sim PY, Phatak S, Moraes G, Wu CY, Cheung CMG, et al

[B2] Yamashiro K, Yanagi Y, Koizumi H, Matsumoto H, Cheung CMG, Gomi F, et al (2022). Relationship between pachychoroid and polypoidal choroidal vasculopathy. J Clin Med.

[B3] Cheung CMG, Lee WK, Koizumi H, Dansingani K, Lai TYY, Freund KB (2019). Pachychoroid disease. Eye.

[B4] Chang YC, Cheng CK (2020). Difference between pachychoroid and nonpachychoroid polypoidal choroidal vasculopathy and their response to anti-vascular endothelial growth factor therapy. Retina.

[B5] Vadalà M, Castellucci M, Guarrasi G, Cillino G, Bonfiglio VME, Casuccio A, et al (2022). Polypoidal choroidal vasculopathy in pachychoroid: Combined treatment with photodynamic therapy and aflibercept. Int Ophthalmol.

[B6] Shimizu Y, Miyata M, Ooto S, Miyake M, Mori Y, Tamura H, et al

[B7] Manayath GJ, Balan R, Ranjan R, Saravanan VR, Narendran V (2022). Posterior choroidal fluid loculation in central serous chorioretinopathy presenting as a choroidal elevation: A rare pachychoroid phenotype. Eur J Ophthalmol.

[B8] Spaide RF, Gemmy Cheung CM, Matsumoto H, Kishi S, Boon CJF, van Dijk EHC, et al (2022). Venous overload choroidopathy: A hypothetical framework for central serous chorioretinopathy and allied disorders. Prog Retin Eye Res.

[B9] Hanumunthadu D, Tan ACS, Singh SR, Sahu NK, Chhablani J (2018). Management of chronic central serous chorioretinopathy. Indian J Ophthalmol.

[B10] Kumar Sahoo N, Lupidi M, Goud A, Gangakhedkar S, Cardillo Piccolino F, Chhablani J (2022). One-year outcome of cystoid macular degeneration in central serous chorioretinopathy. Eur J Ophthalmol.

[B11] Kretz FT, Beger I, Koch F, Nowomiejska K, Auffarth GU, Koss MJ (2015). Randomized clinical trial to compare micropulse photocoagulation versus half-dose verteporfin photodynamic therapy in the treatment of central serous chorioretinopathy. Ophthalmic Surg Lasers Imaging Retina.

[B12] Chan WM, Lai TY, Lai RY, Tang EW, Liu DT, Lam DS (2008). Safety enhanced photodynamic therapy for chronic central serous chorioretinopathy: One-year results of a prospective study. Retina.

[B13] Bousquet E, Beydoun T, Rothschild PR, Bergin C, Zhao M, Batista R, et al (2015). Spironolactone for nonresolving central serous chorioretinopathy: A randomized controlled crossover study. Retina.

[B14] Schwartz R, Habot‐Wilner Z, Martinez MR, Nutman A, Goldenberg D, Cohen S, et al

[B15] Chung YR, Seo EJ, Lew HM, Lee KH (2013). Lack of positive effect of intravitreal bevacizumab in central serous chorioretinopathy: Meta-analysis and review. Eye.

[B16] Ji S, Wei Y, Chen J, Tang S (2017). Clinical efficacy of anti-VEGF medications for central serous chorioretinopathy: A meta-analysis. Int J Clin Pharm.

[B17] Karacorlu M, Ersoz MG, Arf S, Hocaoglu M, Sayman Muslubas I (2018). Long-term follow-up of pachychoroid pigment epitheliopathy and lesion characteristics. Graefes Arch Clin Exp Ophthalmol.

[B18] Sakurada Y, Fragiotta S, Leong BCS, Parikh R, Hussnain SA, Freund KB (2020). Relationship between choroidal vascular hyperpermeability, choriocapillaris flow density, and choroidal thickness in eyes with pachychoroid pigment epitheliopathy. Retina.

[B19] Tagawa M, Ooto S, Yamashiro K, Tamura H, Oishi A, Uji A, et al (2022). Choriocapillaris flow deficit in a pachychoroid spectrum disease using en face optical coherence tomography angiography averaging. Plos One.

[B20] Sagar P, Sodhi PS, Roy S, Takkar B, Azad SV

[B21] Demirel S, Yanık Ö, Özcan G, Batıoğlu F, Özmert E (2021). A comparative study on the choroidal vascularity index and the determination of cut-off values in the pachychoroid spectrum diseases. Jpn J Ophthalmol.

[B22] Lee M, Lee H, Kim HC, Chung H (2018). Changes in Stromal and luminal areas of the choroid in pachychoroid diseases: Insights into the pathophysiology of pachychoroid diseases. Invest Ophthalmol Vis Sci.

[B23] Matsumoto H, Kishi S, Mukai R, Akiyama H (2019). Remodeling of macular vortex veins in pachychoroid neovasculopathy. Sci Rep.

[B24] Fung AT, Yannuzzi LA, Freund KB (2012). Type 1 (sub-retinal pigment epithelial) neovascularization in central serous chorioretinopathy masquerading as neovascular age-related macular degeneration. Retina.

[B25] Cho SC, Ryoo NK, Ahn J, Woo SJ, Park KH (2020). Association of irregular pigment epithelial detachment in central serous chorioretinopathy with genetic variants implicated in age-related macular degeneration. Sci Rep.

[B26] Miyake M, Ooto S, Yamashiro K, Takahashi A, Yoshikawa M, Akagi-Kurashige Y, et al (2015). Pachychoroid neovasculopathy and age-related macular degeneration. Sci Rep.

[B27] Hosoda Y, Miyake M, Yamashiro K, Ooto S, Takahashi A, Oishi A, et al (2020). Deep phenotype unsupervised machine learning revealed the significance of pachychoroid features in etiology and visual prognosis of age-related macular degeneration. Sci Rep.

[B28] Kim YH, Lee B, Kang E, Oh J (2021). Clustering of eyes with age-related macular degeneration or pachychoroid spectrum diseases based on choroidal thickness profile. Sci Rep.

[B29] Terao N, Koizumi H, Kojima K, Yamagishi T, Yamamoto Y, Yoshii K, et al (2018). Distinct aqueous humour cytokine profiles of patients with pachychoroid neovasculopathy and neovascular age-related macular degeneration. Sci Rep.

[B30] Hata M, Yamashiro K, Ooto S, Oishi A, Tamura H, Miyata M, et al (2017). Intraocular vascular endothelial growth factor levels in pachychoroid neovasculopathy and neovascular age-related macular degeneration. Invest Ophthalmol Vis Sci.

[B31] Yamada C, Mukai R, Shinohara Y, Matsumoto H, Akiyama H (2022). Occlusion of a vortex vein after treatment with half-fluence photodynamic therapy combined with intravitreal aflibercept injection for pachychoroid neovasculopathy. Cureus.

[B32] Su Y, Zhang X, Gan Y, Zeng Y, Wen F (2022). Detection of pachychoroid neovasculopathy with optical coherence tomography angiography versus dye angiography imaging. Photodiagnosis Photodyn Ther.

[B33] Dansingani KK, Balaratnasingam C, Klufas MA, Sarraf D, Freund KB (2015). Optical coherence tomography angiography of shallow irregular pigment epithelial detachments in pachychoroid spectrum disease. Am J Ophthalmol.

[B34] Jung BJ, Kim JY, Lee JH, Baek J, Lee K, Lee WK (2019). Intravitreal aflibercept and ranibizumab for pachychoroid neovasculopathy. Sci Rep.

[B35] Padrón-Pérez N, Arias L, Rubio M, Lorenzo D, García-Bru P, Català-Mora J, et al (2018). Changes in choroidal thickness after intravitreal injection of anti-vascular endothelial growth factor in pachychoroid neovasculopathy. Invest Ophthalmol Vis Sci.

[B36] Lee JH, Lee WK (2016). One-year results of adjunctive photodynamic therapy for type 1 neovascularization associated with thickened choroid. Retina.

[B37] Yanık Ö, Demirel S, Batıoğlu F, Özmert E (2022). A comparative study of short-term vascular and stromal alterations of the choroid following half-fluence photodynamic therapy in pachychoroid neovasculopathy and chronic central serous chorioretinopathy. Life.

[B38] Kitajima Y, Maruyama-Inoue M, Ito A, Sato S, Inoue T, Yamane S, et al (2020). One-year outcome of combination therapy with intravitreal anti-vascular endothelial growth factor and photodynamic therapy in patients with pachychoroid neovasculopathy. Graefes Arch Clin Exp Ophthalmol.

[B39] Matsumoto H, Mukai R, Kikuchi Y, Morimoto M, Akiyama H (2020). One-year outcomes of half-fluence photodynamic therapy combined with intravitreal injection of aflibercept for pachychoroid neovasculopathy without polypoidal lesions. Jnp J Ophthalmol.

[B40] Tsujikawa A, Sasahara M, Otani A, Gotoh N, Kameda T, Iwama D, et al (2007). Pigment epithelial detachment in polypoidal choroidal vasculopathy. Am J Ophthalmol.

[B41] Pereira A, Aldrees S, Pimentel MC, Yan P

[B42] Demirel S, Güran Beğar P, Yanık Ö, Batıoğlu F, Özmert E (2022). Visualization of type-1 macular neovascularization secondary to pachychoroid spectrum diseases: A Comparative study for sensitivity and specificity of indocyanine green angiography and optical coherence tomography angiography. Diagnostics.

[B43] Kishi S, Matsumoto H (2022). A new insight into pachychoroid diseases: Remodeling of choroidal vasculature. Graefes Arch Clin Exp Ophthalmol.

[B44] Zhan Z, Sun L, Jin C, Yang Y, Hu A, Tang M, et al (2019). Comparison between non-visualized polyps and visualized polyps on optical coherence tomography angiography in polypoidal choroidal vasculopathy. Graefes Arch Clin Exp Ophthalmol.

[B45] Dansingani KK, Gal-Or O, Sadda SR, Yannuzzi LA, Freund KB (2018). Understanding aneurysmal type 1 neovascularization (polypoidal choroidal vasculopathy): A lesson in the taxonomy of 'expanded spectra' - A review. Clin Exp Ophthalmol.

[B46] Siedlecki J, Klaas JE, Keidel LF, Asani B, Luft N, Priglinger SG, et al (2022). Progression of pachychoroid neovasculopathy into aneurysmal type 1 choroidal neovascularization or polypoidal choroidal vasculopathy. Ophthalmol Retina.

[B47] Bakthavatsalam M, Ng DS, Lai FH, Tang FY, Brelén ME, Tsang CW, et al (2017). Choroidal structures in polypoidal choroidal vasculopathy, neovascular age-related maculopathy, and healthy eyes determined by binarization of swept source optical coherence tomographic images. Graefes Arch Clin Exp Ophthalmol.

[B48] Fan Q, Cheung CMG, Chen LJ, Yamashiro K, Ahn J, Laude A, et al (2017). Shared genetic variants for polypoidal choroidal vasculopathy and typical neovascular age-related macular degeneration in East Asians. J Hum Genet.

[B49] Baek J, Lee JH, Jung BJ, Kook L, Lee WK (2018). Morphologic features of large choroidal vessel layer: Age-related macular degeneration, polypoidal choroidal vasculopathy, and central serous chorioretinopathy. Graefes Arch Clin Exp Ophthalmol.

[B50] Wang W, He M, Zhang X (2014). Combined intravitreal anti-VEGF and photodynamic therapy versus photodynamic monotherapy for polypoidal choroidal vasculopathy: A systematic review and meta-analysis of comparative studies. PLoS One.

[B51] Sakurada Y, Sugiyama A, Tanabe N, Kikushima W, Kume A, Iijima H (2017). Choroidal thickness as a prognostic factor of photodynamic therapy with aflibercept or ranibizumab for polypoidal choroidal vasculopathy. Retina.

[B52] Montorio D, Giordano M, Concilio M, Cennamo G (2022). Structural and vascular changes of the choroid in polypoidal choroidal vasculopathy after intravitreal anti-vegf therapy. Ophthalmologica.

[B53] Kumawat D, Bhayana A, Kumar V (2019). Pachychoroid spectrum disorders: A review of clinical features and management. DJO.

[B54] Lee WK, Baek J, Dansingani KK, Lee JH, Freund KB

[B55] Hubschman S, Hou K, Sarraf D, Tsui I (2022). An unusual presentation of peripapillary pachychoroid syndrome. Am J Ophthalmol Case Rep.

[B56] Phasukkijwatana N, Freund KB, Dolz-Marco R, Al-Sheikh M, Keane PA, Egan CA, et al (2018). Peripapillary pachychoroid syndrome. Retina.

[B57] Xu D, Garg E, Lee K, Sakurada Y, Amphornphruet A, Phasukkijwatana N, et al (2022). Long-term visual and anatomic outcomes of patients with peripapillary pachychoroid syndrome. Br J Ophthalmol.

[B58] Iovino C, Peiretti E, Tatti F, Querques G, Borrelli E, Sacconi R, et al (2022). Photodynamic therapy as a treatment option for peripapillary pachychoroid syndrome: A pilot study. Eye.

[B59] Barequet D, Iglicki M, Meshi A, Loewenstein A, Goldstein M, Zur D (2022). Acquired vitelliform lesions: A novel finding in eyes with peripapillary pachychoroid syndrome. Retina.

[B60] Bouzika P, Georgalas I, Sotirianakou ME, Karamaounas A, Symeonidis C, Tyrlis K, et al (2022). Peripapillary pachychoroid syndrome (PPS): Diagnosing and treating a rare entity. Case Rep Ophthalmol Med.

[B61] Sen P, Sreenivasan J, Maitra P (2021). A case of peripapillary pachychoroid syndrome treated with anti-vascular endothelial growth factor injections. Indian J Ophthalmol-Case Rep.

[B62] Alonso-Martín B, de-Lucas-Viejo B, Gimeno-Carrero M, Ferro-Osuna M, Sambricio J (2020). Diagnosis by multimodal imaging in peripapillary pachychoroid syndrome: A case report. Arch Soc Esp Oftalmol.

[B63] Manayath GJ, Verghese S, Ranjan R, Narendran V (2022). Photodynamic therapy for peripapillary pachychoroid syndrome-a case report. Digit J Ophthalmol.

[B64] Mohabati D, Hoyng CB, Yzer S, Boon CJF (2020). Clinical characteristics and outcome of posterior cystoid macular degeneration in chronic central serous chorioretinopathy. Retina.

[B65] Yzer S, Pothof A, Martinez J, Behar-Cohen FF

[B66] Pothof AB, Fernández-Avellaneda P, Behar-Cohen F, Ciriano JPM, Yzer S

[B67] Verma S, Kumar V, Azad S, Bhayana AA, Surve A, Kumar S, et al (2021). Focal choroidal excavation: Review of literature. Br J Ophthalmol.

[B68] Chung H, Byeon SH, Freund KB (2017). Difference between pachychoroid and nonpachychoroid polypoidal choroidal vasculopathy and their response to anti-vascular endothelial growth factor therapy. Retina.

[B69] Ellabban AA, Tsujikawa A, Ooto S, Yamashiro K, Oishi A, Nakata I, et al (2013). Focal choroidal excavation in eyes with central serous chorioretinopathy. Am J Ophthalmol.

[B70] Lim FP, Wong CW, Loh BK, Chan CM, Yeo I, Lee SY, et al (2016). Prevalence and clinical correlates of focal choroidal excavation in eyes with age-related macular degeneration, polypoidal choroidal vasculopathy and central serous chorioretinopathy. Br J Ophthalmol.

[B71] Shinojima A, Kawamura A, Mori R, Yuzawa M (2014). Morphologic features of focal choroidal excavation on spectral domain optical coherence tomography with simultaneous angiography. Retina.

[B72] Dhodapkar RM, Spadaro JZ, Adelman RA (2022). A case of extrafoveal focal choroidal excavation. Am J Ophthalmol Case Rep.

[B73] Seo EJ, Moon TH, Kim DY, Chae JB (2021). Choroidal inflammation and choriocapillaris ischemia in focal choroidal excavation in comparison to pachychoroid nevascularopathy. Retina.

[B74] Margolis R, Mukkamala SK, Jampol LM, Spaide RF, Ober MD, Sorenson JA, et al (2011). The expanded spectrum of focal choroidal excavation. Arch Ophthalmol.

[B75] Capellan P, Gonzalez LA, Abdallah Mahrous M, Weiss SJ, Botsford B, Lenis TL, et al (2021). Primary and secondary focal choroidal excavation morphologic phenotypes, associated ocular disorders and prognostic implications. Br J Ophthalmol.

[B76] Wakabayashi Y, Nishimura A, Higashide T, Ijiri S, Sugiyama K

[B77] Kumano Y, Nagai H, Enaida H, Ueno A, Matsui T

[B78] Gan Y, Ji Y, Zuo C, Su Y, Liao N, Zhang X, et al (2022). Correlation between focal choroidal excavation and underlying retinochoroidal disease: A pathological hypothesis from clinical observation. Retina.

[B79] Montero Hernández J, Remolí Sargues L, Monferrer Adsuara C, Castro Navarro V, Navarro Palop C, Cervera Taulet E

[B80] Shroff D, Sharma M, Chhablani J, Gupta P, Gupta C, Shroff C

[B81] Mantel I, Schalenbourg A, Zografos L (2012). Peripheral exudative hemorrhagic chorioretinopathy: Polypoidal choroidal vasculopathy and hemodynamic modifications. Am J Ophthalmol.

[B82] Goldman DR, Freund KB, McCannel CA, Sarraf D (2013). Peripheral polypoidal choroidal vasculopathy as a cause of peripheral exudative hemorrhagic chorioretinopathy: A report of 10 eyes. Retina.

[B83] Izumi T, Maruko I, Kawano T, Sakaihara M, Iida T (2022). Morphological differences of choroid in central serous chorioretinopathy determined by ultra-widefield optical coherence tomography. Graefes Arch Clin Exp Ophthalmol.

[B84] Branchini LA, Adhi M, Regatieri CV, Nandakumar N, Liu JJ, Laver N, et al (2013). Analysis of choroidal morphologic features and vasculature in healthy eyes using spectral-domain optical coherence tomography. Ophthalmology.

[B85] Pinarci EY, Kilic I, Bayar SA, Sizmaz S, Akkoyun I, Yilmaz G (2013). Clinical characteristics of peripheral exudative hemorrhagic chorioretinopathy and its response to bevacizumab therapy. Eye.

[B86] Shields CL, Salazar PF, Mashayekhi A, Shields JA (2009). Peripheral exudative hemorrhagic chorioretinopathy simulating choroidal melanoma in 173 eyes. Ophthalmology.

[B87] Singh RB, Perepelkina T, Testi I, Young BK, Mirza T, Invernizzi A, et al

[B88] Dansingani KK, Balaratnasingam C, Naysan J, Freund KB (2016). En face imaging of pachychoroid spectrum disorders with swept-source optical coherence tomography. Retina.

[B89] Spaide RF, Ledesma-Gil G, Gemmy Cheung CM (2021). Intervortex venous anastomosis in pachychoroid-related disorders. Retina.

[B90] Brinks J, van Dijk EHC, Meijer OC, Schlingemann RO, Boon CJF (2022). Choroidal arteriovenous anastomoses: A hypothesis for the pathogenesis of central serous chorioretinopathy and other pachychoroid disease spectrum abnormalities. Acta Ophthalmol.

[B91] Schrödl F, Kaser-Eichberger A, Trost A, Strohmaier C, Bogner B, Runge C, et al (2015). Lymphatic markers in the adult human choroid. Invest Ophthalmol Vis Sci.

[B92] Spaide RF, Ledesma-Gil G (2021). Choriocapillaris vascular parameters in normal eyes and those with pachychoroid with and without disease. Retina.

[B93] Jeong S, Kang W, Noh D, van Hemert J, Sagong M (2022). Choroidal vascular alterations evaluated by ultra-widefield indocyanine green angiography in central serous chorioretinopathy. Graefes Arch Clin Exp Ophthalmol.

[B94] Bacci T, Oh DJ, Singer M, Sadda S, Freund KB (2022). Ultra-widefield indocyanine green angiography reveals patterns of choroidal venous insufficiency influencing pachychoroid disease. Invest Ophthalmol Vis Sci.

[B95] Imanaga N, Terao N, Nakamine S, Tamashiro T, Wakugawa S, Sawaguchi K, et al (2021). Scleral thickness in central serous chorioretinopathy. Ophthalmol Retina.

[B96] Buckhurst HD, Gilmartin B, Cubbidge RP, Logan NS (2015). Measurement of scleral thickness in humans using anterior segment optical coherent tomography. PLoS One.

[B97] Dhakal R, Vupparaboina KK, Verkicharla PK (2020). Anterior sclera undergoes thinning with increasing degree of myopia. Invest Ophthalmol Vis Sci.

[B98] Terao N, Koizumi H, Kojima K, Kusada N, Nagata K, Yamagishi T, et al (2020). Short axial length and hyperopic refractive error are risk factors of central serous chorioretinopathy. Br J Ophthalmol.

[B99] Kishi S, Matsumoto H, Sonoda S, Hiroe T, Sakamoto T, Akiyama H (2018). Geographic filling delay of the choriocapillaris in the region of dilated asymmetric vortex veins in central serous chorioretinopathy. PLoS One.

[B100] Matsumoto H, Hoshino J, Mukai R, Nakamura K, Kikuchi Y, Kishi S, et al (2020). Vortex vein anastomosis at the watershed in pachychoroid spectrum diseases. Ophthalmol Retina.

[B101] Chen LL, Wang Q, Yu WH, Chen YX (2018). Choroid changes in vortex vein-occluded monkeys. Int J Ophthalmol.

[B102] Shinojima A, Mehanna C, Lavia CA, Gaudric A, Tadayoni R, Bousquet E (2020). Central serous chorioretinopathy: Risk factors for serous retinal detachment in fellow eyes. Br J Ophthalmol.

[B103] Kunikata H, Sato R, Nishiguchi KM, Nakazawa T (2020). Systemic oxidative stress level in patients with central serous chorioretinopathy. Graefes Arch Clin Exp Ophthalmol.

[B104] Kim YH, Kang E, Oh J (2022). Factors related to the location of pigment epithelial detachment in central serous chorioretinopathy. Sci Rep.

[B105] Yagi M, Miyake M, Mori Y, Hosoda Y, Takahashi A, Muraoka Y, et al (2022). Natural course of pachychoroid pigment epitheliopathy. Ophthalmol Sci.

[B106] Pang CE, Freund KB (2015). Pachychoroid neovasculopathy. Retina.

[B107] Tang J, Han X, Tang R, Li M, Wang Z, Zhao M, et al (2022). Case series: Pachychoroid pigment epitheliopathy transformed to polypoidal choroidal vasculopathy after long-term follow-up. BMC Ophthalmol.

[B108] Gerardy M, Yesilirmak N, Legras R, Behar-Cohen F, Bousquet E (2022). Central serous chorioretinopathy: High-resolution imaging of asymptomatic fellow eyes using adaptive optics scanning laser ophthalmoscopy. Retina.

[B109] Castro-Navarro V, Behar-Cohen F, Chang W, Joussen AM, Lai TYY, Navarro R, et al (2021). Pachychoroid: Current concepts on clinical features and pathogenesis. Graefes Arch Clin Exp Ophthalmol.

[B110] Imamura Y, Fujiwara T, Margolis R, Spaide RF (2009). Enhanced depth imaging optical coherence tomography of the choroid in central serous chorioretinopathy. Retina.

[B111] Izumi T, Koizumi H, Maruko I, Takahashi Y, Sonoda S, Sakamoto T, et al (2017). Structural analyses of choroid after half-dose verteporfin photodynamic therapy for central serous chorioretinopathy. Br J Ophthalmol.

[B112] Iu LPL, Chan HY, Ho M, Lai FHP, Mak ACY, Wong RLM, et al (2021). The contemporary role of photodynamic therapy in the treatment of pachychoroid diseases. J Ophthalmol.

[B113] Yannuzzi LA (1987). Type-A behavior and central serous chorioretinopathy. Retina.

[B114] Conrad R, Geiser F, Kleiman A, Zur B, Karpawitz-Godt A (2014). Temperament and character personality profile and illness-related stress in central serous chorioretinopathy. SciWorldJ.

[B115] Sahin A, Bez Y, Kaya MC, Türkcü FM, Sahin M, Yüksel H (2014). Psychological distress and poor quality of life in patients with central serous chorioretinopathy. Semin Ophthalmol.

[B116] Cheong KX, Barathi VA, Teo KYC, Chakravarthy U, Tun SBB, Busoy JM, et al (2021). Choroidal and retinal changes after systemic adrenaline and photodynamic therapy in non-human primates. Invest Ophthalmol Vis Sci.

[B117] Maltsev DS, Kulikov AN, Vasiliev AS, Chhablani J (2022). Accommodation is decreased in eyes with acute central serous chorioretinopathy. Optom Vis Sci.

[B118] Maltsev DS, Kulikov AN, Vasiliev AS (2020). Effect of topical pilocarpine on choroidal thickness in healthy subjects. Optom Vis Sci.

[B119] Yeung SC, Park JY, Park D, You Y, Yan P (2022). The effect of systemic and topical ophthalmic medications on choroidal thickness: A review. Br J Clin Pharmacol.

[B120] Singh SR, Vupparaboina KK, Goud A, Dansingani KK, Chhablani J (2019). Choroidal imaging biomarkers. Surv Ophthalmol.

[B121] Agrawal R, Chhablani J, Tan KA, Shah S, Sarvaiya C, Banker A (2016). Choroidal vascularity index in central serous chorioretinopathy. Retina.

[B122] Agrawal R, Gupta P, Tan K-A, Cheung CMG, Wong T-Y, Cheng C-Y (2016). Choroidal vascularity index as a measure of vascular status of the choroid: Measurements in healthy eyes from a population-based study. Sci Rep.

[B123] Mirshahi R, Naseripour M, Shojaei A, Heirani M, Alemzadeh SA, Moodi F, et al (2022). Differentiating a pachychoroid and healthy choroid using an unsupervised machine learning approach. Sci Rep.

